# Differential Phospho‐Signatures in Blood Cells Identify *LRRK2* G2019S Carriers in Parkinson's Disease

**DOI:** 10.1002/mds.28927

**Published:** 2022-01-20

**Authors:** Alicia Garrido, Enrique Santamaría, Joaquín Fernández‐Irigoyen, Marta Soto, Cristina Simonet, Manel Fernández, Donina Obiang, Eduardo Tolosa, María‐José Martí, Shalini Padmanabhan, Cristina Malagelada, Mario Ezquerra, Rubén Fernández‐Santiago

**Affiliations:** ^1^ Parkinson Disease and Movement Disorders Unit, Neurology Service Institut Clínic de Neurociències, Hospital Clínic de Barcelona Barcelona Catalonia Spain; ^2^ Lab of Parkinson Disease & Other Neurodegenerative Movement Disorders Institut d'Investigacions Biomèdiques August Pi i Sunyer (IDIBAPS), Institut de Neurociències, Universitat de Barcelona Barcelona Catalonia Spain; ^3^ Centro de Investigación Biomédica en Red sobre Enfermedades Neurodegenerativas (CIBERNED: CB06/05/0018‐ISCIII) Barcelona Catalonia Spain; ^4^ Proteored‐ISCIII, Proteomics Platform, Clinical Neuroproteomics Unit, Navarrabiomed, Departamento de Salud, UPNA, IdiSNA Pamplona Navarra Spain; ^5^ Parkinson's Disease and Movement Disorders Group of the Institut de Neurociències (Universitat de Barcelona) Barcelona Catalonia Spain; ^6^ The Michael J. Fox Foundation for Parkinson's Research, Grand Central Station New York New York USA; ^7^ Department of Biomedicine, Faculty of Medicine Universitat de Barcelona Barcelona Catalonia Spain; ^8^ Institut de Neurociències Universitat de Barcelona Barcelona Catalonia Spain; ^9^ Histology Unit, Department of Biomedicine, Faculty of Medicine Universitat de Barcelona Barcelona Catalonia Spain

**Keywords:** biomarkers, leucine‐rich repeat kinase 2 (LRRK2), nonmanifesting carriers, Parkinson's disease (PD), peripheral blood mononuclear cells (PBMCs), phospho‐proteomics

## Abstract

**Background:**

The clinicopathological phenotype of G2019S LRRK2‐associated Parkinson's disease (L2PD) is similar to idiopathic Parkinson's disease (iPD), and G2019S LRRK2 nonmanifesting carriers (L2NMCs) are at increased risk for development of PD. With various therapeutic strategies in the clinical and preclinical pipeline, there is an urgent need to identify biomarkers that can aid early diagnosis and patient enrichment for ongoing and future LRRK2‐targeted trials.

**Objective:**

The objective of this work was to investigate differential protein and phospho‐protein changes related to G2019S mutant LRRK2 in peripheral blood mononuclear cells from G2019S L2PD patients and G2019S L2NMCs, identify specific phospho‐protein changes associated with the G2019S mutation and with disease status, and compare findings with patients with iPD.

**Methods:**

We performed an unbiased phospho‐proteomic study by isobaric label–based mass spectrometry using peripheral blood mononuclear cell group pools from a LRRK2 cohort from Spain encompassing patients with G2019S L2PD (n = 20), G2019S L2NMCs (n = 20), healthy control subjects (n = 30), patients with iPD (n = 15), patients with R1441G L2PD (n = 5), and R1441G L2NMCs (n = 3) (total N = 93).

**Results:**

Comparing G2019S carriers with healthy controls, we identified phospho‐protein changes associated with the G2019S mutation. Moreover, we uncovered a specific G2019S phospho‐signature that changes with disease status and can discriminate patients with G2019S L2PD, G2019S L2NMCs, and healthy controls. Although patients with iPD showed a differential phospho‐proteomic profile, biological enrichment analyses revealed similar changes in deregulated pathways across the three groups.

**Conclusions:**

We found a differential phospho‐signature associated with LRRK2 G2019S for which, consistent with disease status, the phospho‐profile from PD at‐risk G2019S L2NMCs was more similar to healthy controls than patients with G2019S L2PD with the manifested disease. © 2022 The Authors. *Movement Disorders* published by Wiley Periodicals LLC on behalf of International Parkinson and Movement Disorder Society

## Graphical abstract

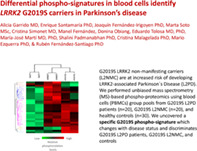

Mutations in the leucine‐rich repeat kinase 2 (*LRRK2*) gene cause autosomal‐dominant LRRK2‐associated Parkinson's disease (L2PD) with a clinical phenotype similar to idiopathic Parkinson's disease (iPD).^1^, ^2^ The G2019S mutation is located within the kinase domain of LRRK2 and represents one of the most frequent genetic causes of PD. It is responsible for about 1% to 2% of the sporadic PD population and 40% in specific ethnic populations.^3^, ^4^, ^5^, ^6^, ^7^ Nonmanifesting carriers of LRRK2 G2019S (L2NMC) or other LRRK2 pathogenic mutations are at a higher risk for PD that increases with age.^1^ However, predicting eventual phenoconversion and disease onset is still challenging^8^ given that the penetrance of G2019S is limited and modulated by additional factors that, beyond advanced age, remain unknown.^9^, ^10^, ^11^, ^12^, ^13^ Moreover, small‐molecule LRRK2 inhibitors,^14^ currently in phase II trials, are a promising therapeutic avenue for L2PD^13^ and possibly also for iPD in which the LRRK2 activity could also be deregulated.^15^, ^16^

As a Ser/Thr kinase, LRRK2 participates in multikinase signaling cascades. In vitro studies showed that pathogenic mutations such as G2019S within the kinase domain or R1441G at the guanosine triphosphatase (GTPase) domain enhance LRRK2 activity,^17^ thus supporting a kinase gain of function in PD.^18^ In 2016, a subset of vesicle trafficking Rab proteins were identified as the first endogenous substrates for LRRK2.^19^ These findings were subsequently validated in other studies.^20^, ^21^ However, confirmation of these findings in large cohorts of patients with L2PD is warranted. The only study available reported that R1441G mutation carriers, but not G2019S mutation carriers, show elevated RAB10 phosphorylation in patients with L2PD.^22^ These results might be related to a higher kinase activity enhancement in R1441G (4‐fold) than G2019S (<2‐fold).^20^, ^23^, ^24^ Moreover, the mutant LRRK2 signaling pathways in patients with G2019S L2PD, its interactors, upstream regulators, and downstream effectors remain widely unknown.^17^, ^25^ Elucidating these molecular players is key to unraveling the disease mechanisms triggered by mutant LRRK2 and identifying disease progression and drug response biomarkers.

Our study aimed to explore protein and phospho‐protein systemic changes related to mutant LRRK2 using accessible blood cells from G2019S carriers and to identify the functional pathways associated with G2019S. Supported by The Michael J. Fox Foundation for Parkinson's Research, we established the Barcelona LRRK2 Biorepository, a collection of peripheral blood mononuclear cells (PBMCs) from G2019S mutation carriers along with detailed clinical data on each enrolled participant. Using this cohort, we performed an unbiased phospho‐proteomic discovery study by isobaric label–based mass spectrometry (MS). In Alzheimer's disease (AD), phospho‐proteome approaches have identified signaling pathways altered during early disease progression,^26^, ^27^ as well as novel tau phosphorylation interactors.^28^ In this study, our core analysis included group pools of PBMC samples from patients with G2019S L2PD (n = 20), G2019S L2NMCs (n = 20), and healthy controls (n = 30). In an exploratory analysis, we also tested patients with iPD (n = 15), patients with R1441G L2PD (n = 5), and R1441G L2NMCs (n = 3). Across pairwise comparisons, we identified group‐specific differentially expressed proteins (DEPs) and differentially phosphorylated proteins (DPPs). Moreover, we found a specific phospho‐protein signature of 23 DPPs, which changes with disease status and can discriminate patients with G2019S L2PD, G2019S L2NMCs, and healthy controls.

## Subjects and Methods

### Subjects

Blood samples from G2019S and R1441G mutation carriers (patients with L2PD and L2NMCs), patients with iPD, and healthy controls were obtained at the Movement Disorders Unit from the Hospital Clínic de Barcelona. Patients with PD were clinically diagnosed according to the UK PD Society Brain Bank criteria, except that more than one affected relative with PD was not an exclusion criterion.^29^ The study included PBMC sample pools from N = 93 subjects consisting of patients with G2019S L2PD (n = 20), G2019S L2NMCs (n = 20), healthy control subjects (n = 30), patients with iPD (n = 15), patients with R1441G L2PD (n = 5), and R1441G L2NMCs (n = 3) (Table 1). All subjects were Caucasians of Spanish descent. Patients with G2019S L2PD were recruited as available (age at sampling, 62.8 ± 11.1 years; disease duration, 8.4 ± 4.3 years; male/female ratio, 1:3) and were matched by age and sex to healthy controls (patient spouses). G2019S L2NMCs (relatives of patients with G2019S L2PD) were recruited according to availability (age at sampling, 52.0 ± 10.1 years) and were 10 years younger than patients with G2019S L2PD. In the complementary analyses, patients with iPD were matched to G2019S L2PD by similar disease duration (7.3 ± 3.4 years), and R1441G carriers who are less frequent in our population were recruited as available. Regarding medication, L2PD as a group (G2019S and R1441G) had a slightly higher levodopa (l‐dopa) equivalent daily dose than patients with iPD (695 vs. 672 mg) and a higher proportion of patients taking l‐dopa (88% vs. 73%). The higher percentage of l‐dopa–treated L2PD patients can be related to their slightly longer evolution time (8.4 ± 4.3 vs. 7.3 ± 3.4 years) and their relatively worse Schwab & England daily activity of 91% (70–100) versus 97% (80–100). Overall, we did not find significant medication differences between L2PD and iPD. The local ethics committee of the Hospital Clínic de Barcelona approved the study, and all subjects gave written informed consent.

**TABLE 1:** Clinic and demographic data of the LRRK2‐associated Parkinson's disease cohort

Study Subjects (total N = 93)	Mean Age ± SD (y)	Sex (males/females)	Disease Duration (y)	Mean MDS UPDRS Part III ± SD	Mean LEDD ± SD (mg)	Mean H&Y ± SD	Mean S&E (min. to max.)
G2019S L2PD (n = 20)	62.8 ± 11.1	5/15	8.4 ± 4.3	23.1 ± 11.5	729.3 ± 386.0	2.0 ± 0.6	91% (70–100)
G2019S L2NMCs (n = 20)	52.0 ± 10.1	10/10	0	1.9 ± 2.5	0	0	100%
Healthy controls (n = 30)	61.9 ± 8.8	8/22	0	2.6 ± 2.4	0	0	100%
iPD (n = 15)	69.2 ± 8.4	8/7	7.3 ± 3.4	21.5 ± 10.4	671.9 ± 413.2	1.9 ± 0.5	97% (80–100)
R1441G L2PD (n = 5)	65.2 ± 14.0	4/1	11.4 ± 7.5	30.2 ± 14.2	513.5 ± 413.2	2.0 ± 0.7	92% (70–100)
R1441G L2NMC (n = 3)	49.1 ± 22.6	2/1	0	2.7 ± 3.1	0	0	100%

SD, standard deviation; MDS UPDRS, Unified Parkinson's Disease Rating Scale scoring of the Movement Disorders Society; LEDD, levodopa equivalent daily dose; H&Y, Hoehn & Yahr progression scoring; S&E, Schwab & England (Activities of Daily Living) scoring; L2PD, LRRK2‐associated Parkinson's disease; L2NMC, LRRK2 nonmanifesting carrier; iPD, idiopathic Parkinson's disease; min., minimum; max., maximum.

### Genotyping

We used TaqMan SNP assays‐on‐demand on a Step One Plus Real‐time PCR System (Life Technologies Inc., Carlsbad, California, United States) to genotype LRRK2 G2019S (#C‐63498123‐10; Thermo Fisher Scientific, Carlsbad, California, United States) and a commercial TaqMan assay for R1441G/C/H as previously described.^30^

### PBMC Isolation

A total of 8 mL of blood was drawn by peripheral vein puncture in fasting. PBMCs were isolated by density gradient using sodium‐citrate collector tubes (BD Vacutainer CPT, Franklin Lakes, New Jersey, United States, #EAN30382903627821) according to the manufacturer's instructions. Dry pellets were flash‐frozen in liquid N_2_ and stored at −80°C until use. The average time between blood collection and flash‐freeze of dry pellets was 4 h, and the average storage period at −80°C was less than 1 year.

### Protein and Phospho‐Protein Sample Preparation

PBMCs from control and parkinsonian groups were homogenized in lysis buffer containing 7 M urea, 2 M thiourea, and 50 mM DTT supplemented with protease (cOmplete Mini; Roche, Basel, Switzerland) and phosphatase inhibitors (PhosSTOP; Roche). Homogenates were spun down at 100,000*g* for 1 hour at 15°C. After precipitation, protein concentration was measured in the supernatants using the Bradford assay kit (Bio‐Rad, Hercules, California, United States). Reduction, alkylation, digestion, and tandem mass‐tag labeling were performed as described in the Supporting Information Methods (Appendix S1).

### Phospho‐Peptide Enrichment and Liquid Chromatography Tandem MS

Enrichment of phosphorylated peptides was performed applying the SIMAC (sequential elution from IMAC) protocol as previously described.^31^ Unbound peptide pools (nonmodified peptides) and phosphorylated fractions were separated by reverse‐phase chromatography using an Eksigent nanoLC ultra 2D pump fitted with a 75‐μm ID column (Eksigent 0.075 × 250) and analyzed using a 5600 Triple‐TOF system (Sciex). For chromatographic and MS parameters, see Supporting Information Methods (Appendix S1).

### Identification of Differential Proteins and Phospho‐Proteins

Raw MS/MS spectra were processed using the MaxQuant software (version 1.5.8.3)^32^ and searched against the UniProt proteome reference for *H. sapiens* (Proteome ID: UP0000056409606, February 2019). The parameters used were as follows: initial maximum precursor (25 ppm), fragment mass deviations (40 ppm), variable modification (methionine oxidation and N‐terminal acetylation) and fixed modification (methyl methanethiosulfonate, MMTS), enzyme (trypsin) with a maximum of 1 missed cleavage, minimum peptide length (7 amino acids), false discovery rate (FDR) for peptide spectrum matches (PSM) and protein identification (1%). Frequently observed laboratory contaminants were removed. More specifically, during protein sample isolation and processing, potential contaminants encompass all subtypes and isoforms of keratins, hair keratins, keratin‐like proteins, and cytoskeletal keratins, which can be ubiquitously present in the skin, dust, clothes, and chemicals, among others. To avoid any potential interference with the PBMC proteomes, we excluded all these contaminants during protein identification, quantitation, and database search phases. To that end, we used the contaminants.fasta file from the Maxquant installation package, which contains the primary sequence data of conventional proteases used in proteomics experiments and all human keratin subtypes. Protein identification was considered valid with at least one unique or “razor” peptide. Protein quantification was calculated using at least two razors + unique peptides. Statistical significance was calculated by a 2‐tailed Student *t* test (*P* < 0.05) in pairwise comparisons and by one‐way ANOVA (*P* < 0.05). For the DPPs, in the analysis of the PBMC phosphorylated fractions, phospho‐serine, phospho‐threonine, and phospho‐tyrosine were chosen as variable modifications for database searching. We used the Perseus software (version 1.5.6.0)^33^ for statistical analysis and data visualization.

### Protein/Phospho‐Protein Functional Analysis

To elucidate the biological functions of DEPs and DPPs identified in patients with G2019S L2PD, G2019S L2NMCs, and patients with iPD, we performed a biological enrichment analysis in Metascape^34^ using default settings (minimum overlap: 3; minimum enrichment: 1.5; *P* < 0.01). As a statistical significance cutoff, we used a false discovery rate multiple testing adjusted *P* < 0.05. We also performed a network analysis of the interlocked DEPs and DPPs identified in patients with G2019S L2PD, G2019S L2NMCs, and patients with iPD in Ingenuity Pathway Analysis v62089861 (release date: February 17, 2021) (www.ingenuity.com)^35^ under default settings. We used only the database information of experimental‐based evidence to be confident about the potentially affected signaling pathways. Ingenuity Pathway Analysis integrates current knowledge on genes, drugs, chemicals, protein families, processes, and pathways based on their interactions and specific functions.

## Results

**FIG 1:**
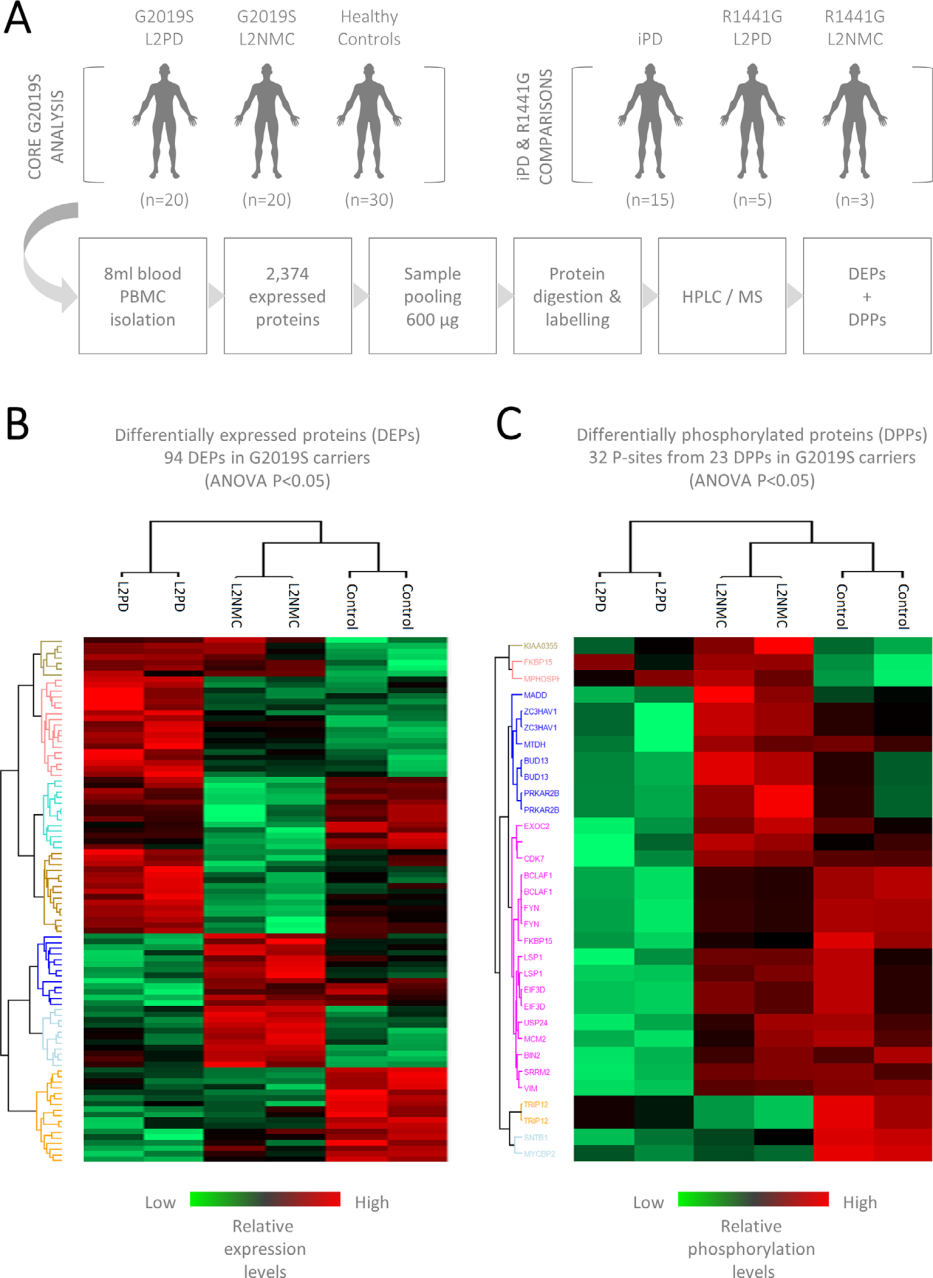
Multigroup comparison of isobaric label–based mass spectrometry (MS) proteomic and phospho‐proteomic data of peripheral blood mononuclear cells (PBMCs) from patients with G2019S LRRK2‐associated Parkinson's disease (L2PD), G2019S LRRK2 nonmanifesting carriers (L2NMCs), and healthy control subjects. (**A**) Experimental design and flowchart. The core analysis included patients with G2019S L2PD, G2019S L2NMCs, and healthy controls. Additional comparative analyses included patients with idiopathic Parkinson's disease (iPD), patients with R1441G L2PD, and R1441G L2NMCs. Prior to isobaric labeling, 600 μg of protein from each subject was pooled in group pools: patients with G2019S L2PD (2 pools, n = 10 subjects each), G2019S L2NMCs (2 pools, n = 10 subjects each), healthy controls (2 pools, n = 15 subjects each), patients with iPD (1 pool, n = 15 subjects), patients with R1441G L2PD (1 pool, n = 5 subjects), and R1441G L2NMCs (1 pool, n = 3 subjects). The total study number was N = 93. (**B**) Differentially expressed proteins (DEPs) in patients with G2019S L2PD, G2019S L2NMCs, and healthy controls by analysis of variance (ANOVA) multigroup comparison (*P* < 0.05). (**C**) Differential phospho‐proteins (DPPs) in patients with G2019S L2PD, G2019S L2NMCs, and healthy controls by ANOVA (*P* < 0.05). Each line corresponds to one differential phospho‐peptide (n = 32) from individual proteins (n = 23). Detected DPPs included ARHGEF4, BCLAF1, BIN2, BUD13, CDK7, EIF3D, EXOC2, FKBP15, FYN, KIAA0355, LSP1, MADD, MCM2, MPHOSPH8, MTDH, MYCBP2, PRKAR2B, SNTB1, SRRM2, TRIP12, USP24, VIM, and ZC3HAV1. HPLC, high‐pressure liquid chromatography. [Color figure can be viewed at wileyonlinelibrary.com]

**FIG 2:**
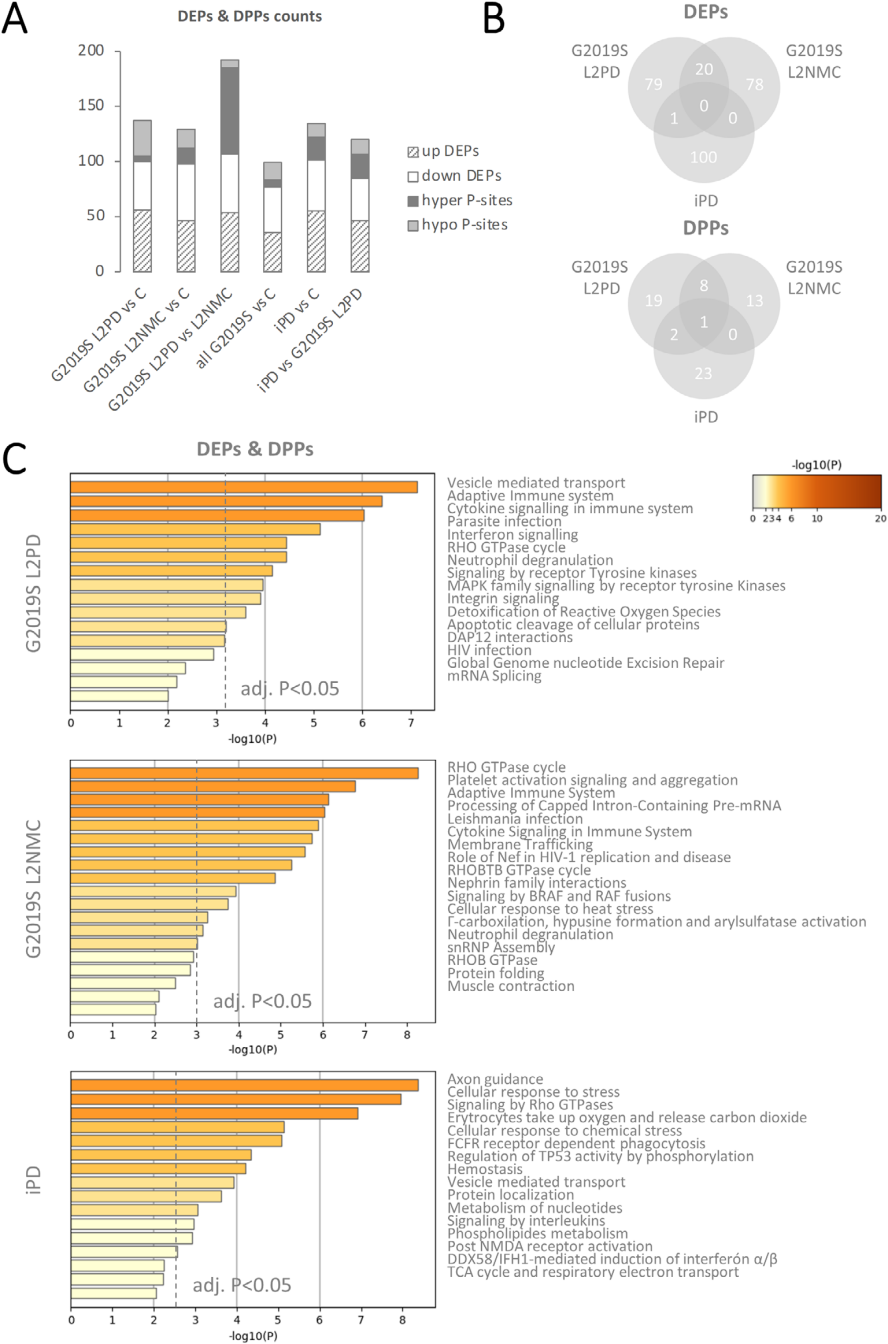
Pairwise comparisons of proteomic and phospho‐proteomic data in LRRK2 G2019S carriers and idiopathic Parkinson's disease (iPD) and biological enrichment analysis. (**A**) Barplot representation of the number of differentially expressed proteins (DEPs; upregulated and downregulated proteins) and differentially phosphorylated proteins (DPPs; hyperphosphorylated and hypophosphorylated peptides) in pairwise comparisons involving G2019S LRRK2‐associated Parkinson's disease (L2PD), G2019S LRRK2 nonmanifesting carrier (L2NMC), and iPD by 2‐tailed Student *t* test (*P* < 0.05). Although the number of DEPs was similar for most of the comparisons, the highest number of phosphorylation differences was observed between G2019S L2PD and G2019S L2NMC. (**B**) Venn diagrams representing the numbers of common and specific DEPs and DPPs showing distinct patterns in G2019S L2PD, G2019S L2NMC, and iPD with respect to healthy controls, yet with a higher degree of overlap between G2019S L2PD and G2019S L2NMC and a poor overlap with iPD. (**C**) Biological enrichment analysis of interlocked DEPs and DPPs in G2019S L2PD, G2019S L2NMC, and iPD compared with healthy controls using Metascape with false discovery rate (FDR) multiple testing adjustment of *P* values (adj. *P* < 0.05) (dashed line). This analysis showed common pathway alteration not only for G2019S carriers but also for iPD. [Color figure can be viewed at wileyonlinelibrary.com]

By unbiased isobaric label‐based MS, we identified 2374 proteins expressed in human PBMCs (Fig. 1A). We first investigated potential phospho‐proteomic changes associated with the LRRK2 G2019S mutation. To this end, we performed pairwise comparisons involving patients with G2019S L2PD, G2019S L2NMCs, and healthy controls by two‐way Student *t* test (*P* < 0.05). Comparing patients with G2019S L2PD with healthy control subjects, we found 100 DEPs and 37 differential phospho‐peptides from 30 DPPs, owing to the fact that phosphorylation at a given protein can occur at one or more Ser/Thr/Tyr residues.^36^ Similarly, in G2019S L2NMCs versus controls, we found 98 DEPs and 31 differential phospho‐sites from 24 DPPs. We also investigated specific proteomic differences in G2019S carriers as a whole, ie, PD and non‐PD, compared with controls and found specific protein differences related to G2019S (77 DEPs; 22 phospho‐sites from 17 DPPs). Moreover, patients with G2019S L2PD and L2NMCs showed the highest phosphorylation differences across all comparisons (107 DEPs; 85 phospho‐sites from 56 DPPs) (Fig. 2A; Supporting Information Tables S1–S4; Supporting Information Fig. S1). Altogether, by comparing LRRK2 G2019S carriers and healthy controls, we identified specific phosphorylation changes associated with the G2019S mutation.

We next inquired whether the phospho‐protein changes in LRRK2 G2019S carriers were associated with changes in disease status. To this end, we performed a multigroup comparison involving all LRRK2 G2019S carriers and healthy controls by one‐way ANOVA (*P* < 0.05). We found 94 DEPs among patients with G2019S L2PD, G2019S L2NMCs, and healthy controls (Fig. 1B). Regarding phospho‐proteins, we found a total of 32 differential phospho‐peptides from 23 DPPs with discriminating features of all three groups (Fig. 1C). This phospho‐signature encompassed the DPPs: ARHGEF4, BCLAF1, BIN2, BUD13, CDK7, EIF3D, EXOC2, FKBP15, FYN, KIAA0355, LSP1, MADD (mitogen‐activated protein kinase [MAPK] activating death domain), MCM2, MPHOSPH8, MTDH, MYCBP2, PRKAR2B, SNTB1, SRRM2, TRIP12, USP24, VIM (Vimentin), and ZC3HAV1 (DEPs and DPPs disclosed for data mining in Supporting Information Tables S5 and S6). Remarkably, consistent with disease status, the phospho‐profile from PD at‐risk G2019S L2NMCs was more similar to controls than patients with G2019S L2PD with the manifested disease. Collectively, these results indicate that G2019S L2NMCs and patients with G2019S L2PD exhibit different phospho‐protein profiles in PBMCs. Accordingly, the identified phospho‐signature can be informative of changes in disease status from asymptomatic to symptomatic G2019S carriers.

Subsequently, we also compared the phospho‐proteome of G2019S carriers and iPD (Fig. 2A). By 2‐tailed Student *t* test (*P* < 0.05), in iPD, we found 101 DEPs and 33 differential phospho‐sites from 26 DPPs with respect to healthy controls. We also found 85 DEPs and 35 phospho‐sites from 23 DPPs in iPD compared with patients with G2019S L2PD (Supporting Information Tables S7 and S8). Overall, most of the DEPs and DPPs in patients with iPD, patients with G2019S L2PD, and G2019S L2NMCs were group specific, yet with a certain degree of overlap only between the two G2019S carrier groups (Fig. 2B). Interestingly, five of the nine DPPs shared by patients with G2019S L2PD and L2NMCs were functionally involved in neuron projection processes (FYN,^37^, ^38^ VIM,^39^ FKBP15,^40^ MYCBP2,^41^, ^42^ and SNTB1^43^, ^44^) (Supporting Information Fig. S2). We also compared the phospho‐proteome of G2019S and R1441G carriers. We found specific protein differences between G2019S L2PD and R1441G L2PD (95 DEPs and 31 phospho‐sites from 25 DPPs) and in R1441G L2PD compared with R1441G L2NMC (89 DEPs and 61 phospho‐peptides from 51 DPPs) (Supporting Information Tables S9 and S10). Yet, given the limited sample size, results involving iPD (n = 15) and R1441G (n = 8) should be regarded as exploratory and need to be interpreted with caution at this stage. Altogether, these results suggest a phospho‐proteomic profile in iPD with only marginal overlap with LRRK2 G2019S carriers, at least in terms of specific DEPs and DPPs.

**FIG 3:**
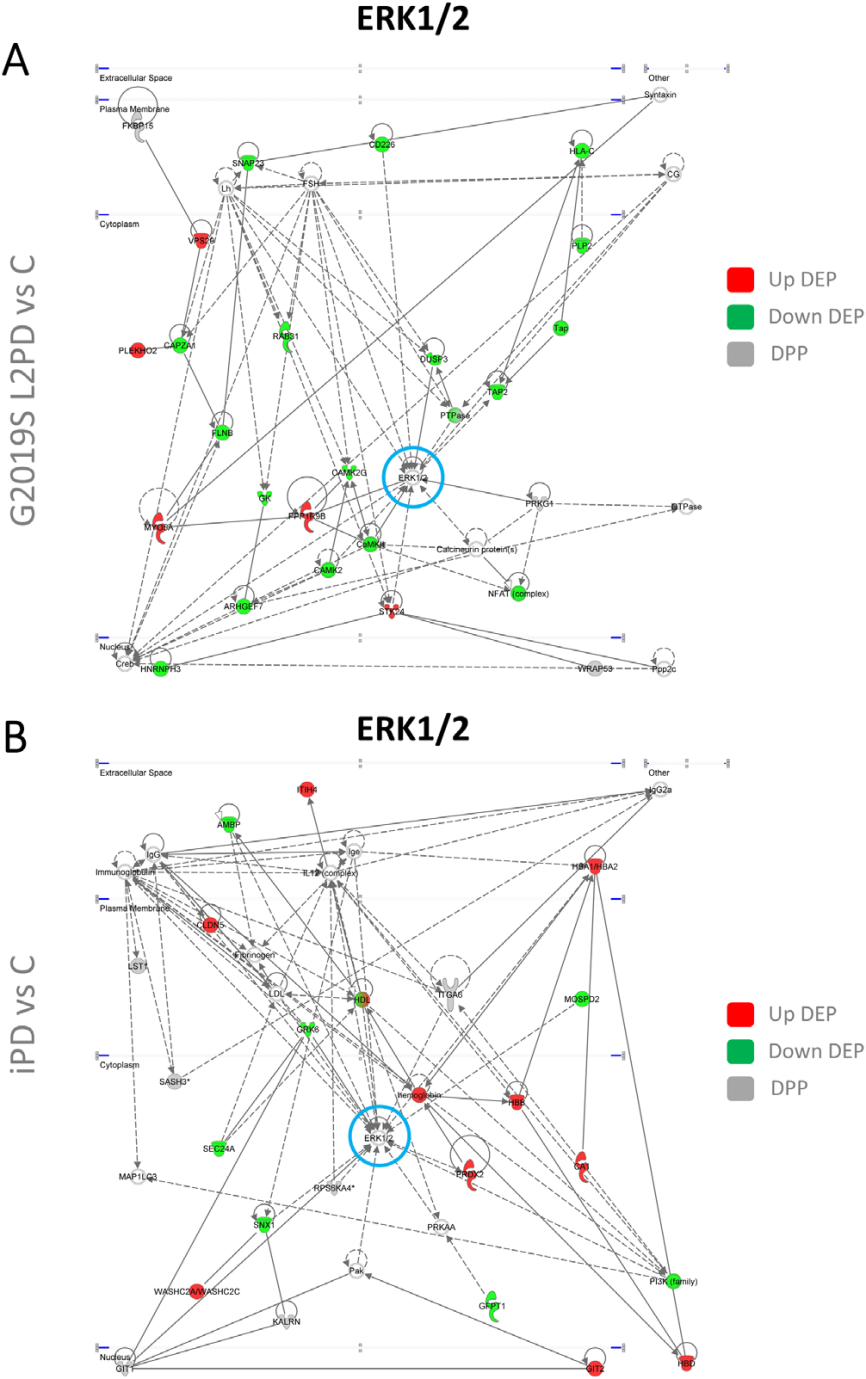
Ingenuity functional network analysis of interlocked differentially expressed proteins (DEPs) and differentially phosphorylated proteins (DPPs) from pairwise comparison showing distinctive networks altered in patients with PD (G2019S LRRK2‐associated Parkinson's disease [L2PD] and idiopathic Parkinson's disease [iPD]). (**A**) Extracellular signal–regulated kinases 1 and 2 (ERK1/2)‐centered network in patients with G2019S L2PD. (**B**) ERK1/2‐centered network in patients with iPD. [Color figure can be viewed at wileyonlinelibrary.com]

**FIG 4:**
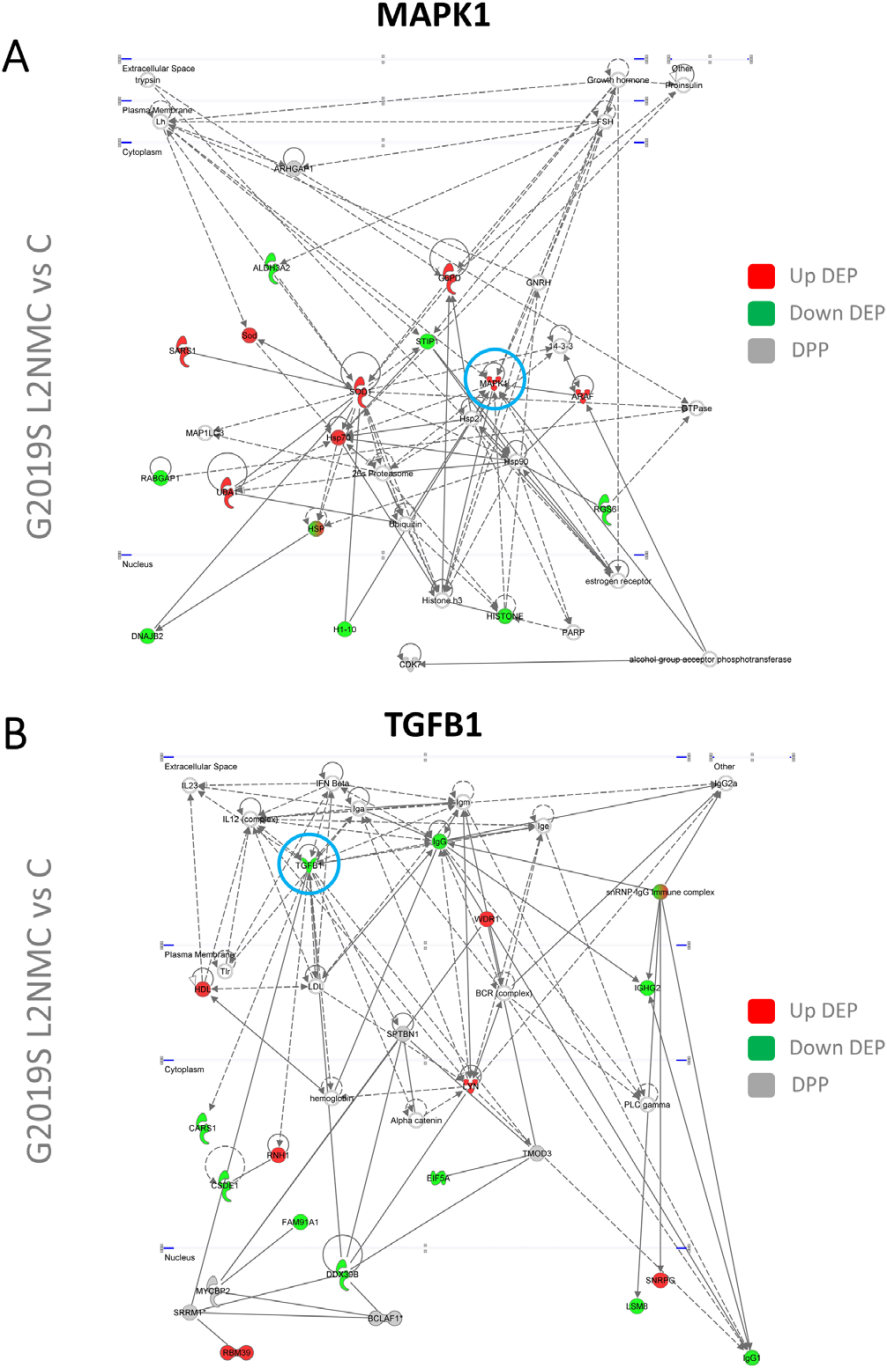
Ingenuity functional network analysis of interlocked DEPs and DPPs from pairwise comparison showing distinctive networks altered in asymptomatic G2019S carriers. (**A**) MAPK1‐centered network in G2019S LRRK2 nonmanifesting carrier (L2NMC), showing upregulated MAPK1. (**B**) Transforming growth factor B1 (TGFB1)‐centered network in G2019S L2NMCs, showing downregulated TGFB1. [Color figure can be viewed at wileyonlinelibrary.com]

Lastly, we performed a biological enrichment analysis to investigate protein functionality at the differential phospho‐signature in G2019S carriers. Using the interlocked 94 DEPs and 23 DPPs, or each separate, we found largely the same Gene Ontology (GO) terms indicating overlapping functions (Supporting Information Fig. S3). The top 3 terms in G2019S carriers included vesicle‐mediated transport, Rho GTPases cycle, and phagocytosis (adjusted *P* < 0.05), which were earlier related to L2PD. RAB regulation of vesicle trafficking was also among the top terms in G2019S carriers. Alternatively, we analyzed the interlocked DEPs and DPPs independently in patients with G2019 L2PD, G2019S L2NMCs, and patients with iPD (Fig. 2). We found similar pathway deregulation despite the marginal DEPs/DPPs overlap between iPD and G2019S carriers. Illustratively, the top term in iPD, axonal outgrowth, is controlled by Rho GTPases.^45^, ^46^, ^47^ To uncover novel protein–protein interaction networks involving DEPs and DPPs identified, we further performed a functional network analysis (adjusted *P* < 0.05). Beyond common network deregulation in iPD, G2019S L2PD, and G2019S L2NMCs (centered around AKT and NFKB) (Supporting Information Fig. S4), we found distinctive networks altered only in G2019S L2PD and iPD (centered around extracellular signal–regulated kinases 1 and 2 [ERK1/2]) (Fig. 3) or only in G2019S L2NMCs (centered around MAPK1 and transforming growth factor Beta 1 [TGFB1]) (Fig. 4).

## Discussion

We performed a phospho‐proteome profiling in PBMCs using a large cohort of LRRK2 G2019S carriers from the Barcelona area in Spain, a mutation representing the most common monogenic cause of familial PD and genetic risk factor of PD in our population.^48^ Beyond in vitro studies,^25^ previous LRRK2 phospho‐proteomic work was done using mice fibroblasts^19^ and neutrophils from modest sets of patients with L2PD.^21^ Such approaches have contributed to identifying novel direct or indirect LRRK2 endogenous substrates.^17^ Yet, there remains a need to identify further other LRRK2 downstream effectors occurring endogenously in biosamples from patients.^17^ In this study, we screened group pooled samples from N = 93 subjects. We quantified 2374 proteins expressed in human PBMCs and identified DEPs and differential phospho‐proteins (DPPs) in G2019S carriers. Moreover, we pinpointed a candidate phospho‐signature encompassing 23 DPPs that discriminates patients with G2019S L2PD, G2019S L2NMCs, and healthy controls.

To the best of our knowledge, this is the first phospho‐proteome study done in PBMCs of LRRK2 carriers. First, when comparing all G2019S carriers with healthy controls, we uncovered specific protein differences associated with the G2019S mutation status (77 DEPs and 17 DPPs). The protein differences in the G2019S L2PD and G2019S L2NMC groups independently were higher than in all G2019S carriers as a whole, thus suggesting additional specific protein phosphorylation differences in PD and non‐PD G2019S carriers. Between G2019S L2PD and G2019S L2NMCs, we found the largest protein phosphorylation differences across all pairwise comparisons (107 DEPs and 56 DPPs). A proteomic study in urine showed DEP changes from G2019S L2NMCs to G2019S L2PD patients.^49^ Similarly, at the phospho‐proteome level, the DPPs identified between G2019S L2PD and L2NMC in our study indicate the presence of specific phosphorylation events associated with changes in disease status.

Second, as a proof of principle, by analysis of variance (ANOVA), we confirmed that the G2019S LRRK2 DPPs change with disease status from asymptomatic G2019S carriers to patients with L2PD with overt disease. Specifically, we uncovered a phospho‐protein signature of 32 phospho‐peptides from 23 unique DPPs with discriminative features to differentiate patients with G2019S L2PD, G2019S L2NMCs, and healthy controls. Consistent with disease status, asymptomatic G2019S L2NMCs were more similar to healthy controls than patients with G2019S L2PD. Because these observations were made in pooled PBMC samples from patients, we cannot establish correlations of DPPs and clinical parameters at the individual level at this time. However, our findings suggest a great potential for unbiased MS‐based approaches in analyzing individual samples^50^ from PD cohorts to establish specific associations between DPPs and clinical parameters (eg, age at onset, Unified Parkinson's Disease Rating Scale Part III scaling, or Montreal Cognitive Assessment scores). Additional phospho‐proteomic initiatives validating our findings can contribute to designing a blood test of early disease detection in G2019S carriers combining clinical and molecular data^51^, ^52^ and finding novel molecular biomarkers for L2PD.^25^ In the absence of early PD detection tests, such novel phospho‐proteomic approaches may hold implications for future PD prediction strategies.^8^, ^53^

The 23 DPPs of the differential phospho‐signature can be plausibly related to mutant LRRK2. Illustratively, MADD is a guanosine triphosphate/guanosine diphosphate exchange factor for RAB proteins.^45^ RABs coordinate interorganellar vesicle‐mediated transport, and some RAB subsets are LRRK2 substrates,^22^ ie, Thr‐73 phospho‐RAB10,^19^, ^20^ a well‐accepted readout of LRRK2 activity.^21^ Although these differences are observed in PBMCs, it is relevant to state that the same proteins play relevant functions in neurons. Thus, downregulation of MADD, a TNF receptor‐binding protein, correlates with neuronal death in Alzheimer's disease.^54^ FYN (Tyrosine‐protein kinase FYN) is associated with PD; it phosphorylates MAP2 and MAPT^55^ and is involved in neural processes by α‐synuclein protein (SNCA) phosphorylation at Tyr‐125.^37^ SNCA oligomers also elicit FYN phosphorylation leading to synaptic dysfunction.^56^ FYN knockdown prevents l‐dopa–induced dyskinesias^57^ and is a therapeutic candidate for PD.^38^ VIM is a cytoskeletal marker for LRRK2 aggregosomes independent of SNCA.^39^ MYCBP2 (MYC binding protein 2) regulates cAMP in the mammalian target of rapamycin pathway controlling axon navigation, synaptogenesis, and axon degeneration in the nervous and olfactory systems.^41^ Through the mammalian target of rapamycin pathway, MYCBP2 controls the transcription of the PD‐associated gene *SYT11*, which along with ATP13A2 regulates autophagy.^42^

Third, we compared the PBMC phospho‐proteome of G2019S carriers and patients with iPD. The differential phospho‐signature was specific for G2019S carriers; however, the biological pathways affected by G2019S‐related DEPs and DPPs overlapped with iPD. These results suggest common disease mechanisms and possible common therapeutic approaches for L2PD and at least some iPD subtypes,^58^ as previously proposed.^13^, ^15^, ^16^ Moreover, in vitro studies showed up to twofold increased kinase activity of LRRK2 G2019S.^17^, ^59^ Yet LRRK2 substrates such as RABs, specifically Thr‐73 RAB10,^19^, ^20^, ^21^ showed increased phosphorylation only in LRRK2 R1441G carriers, but no effect for G2019S.^22^ In the differential phospho‐signature of 23 DPPs, we found more hyperphosphorylated proteins in G2019S L2NMC than in L2PD. Comparing both, most DPPs were hyperphosphorylated in G2019S L2NMC, ie, 78 of 85 phospho‐sites (92%) and 51 of 56 DPPs (91%). One hypothesis is that the G2019S hyperkinase activity would be key to initiating PD mechanisms, highest in L2NMC (eg, as shown using LRRKtide),^59^ but attenuated in L2PD after the onset of the motor symptoms. If valid, initiation of LRRK2 inhibitors treatment in L2NMC before motor manifestation would be an option to investigate in clinical trials.^58^

Functionally, DEPs and DPPs at the G2019S phospho‐signature were involved in vesicle‐mediated transport, Rho GTPases, and phagocytosis, among other terms related to LRRK2 mutations.^17^, ^25^, ^60^, ^61^, ^62^, ^63^ RAB vesicle trafficking was also affected in G2019S carriers, but despite detecting RABs expression in PBMCs, we found no differential RAB phosphorylation. RAB10 Thr‐73–increased phosphorylation was shown as a biomarker for R1441G, but not G2019S, in LRRK2 neutrophils.^21^, ^22^ Moreover, iPD pathways overlapped with G2019S carriers, with axonal outgrowth being the top term, which is controlled by Rho GTPases.^45^, ^46^, ^47^ A previous proteome urine study quantified more than 2000 proteins in G2019S carriers and patients with iPD and found discriminant DEPs profiles for familial PD related to lysosomal dysfunction.^49^ DEPs from another study in G2019S L2PD iPSC‐derived neurons and brain affected the related endocytic pathway.^64^ Lastly, functional network analysis of DEPs and DPPs revealed deregulation of the ERK1/2 survival pathway in patients (G2019S L2PD and iPD)^65^, ^66^ and networks involving MAPK1 (ERK2) and TGFB1^67^ in G2019S L2NMC only. These findings show LRRK2 signaling networks in PBMCs that may be helpful for future biomarker research in L2NMC before motor manifestation.

Interestingly, several of the DPPs identified in our study were also described in a phospho‐proteomic screening of PBMCs from healthy controls treated with the Lu AF58786 LRRK2 and PFE‐360 inhibitors (145 DPPs).^68^ Common DPPs included BIN2, CEP170, NUCKS1, and SRRM2 from L2PD (4/30); ATG9A, LRRK2, and ZC3HAV1 from L2NMC (3/24); and RBM33, RCSD1, and TJP2 from iPD (4/30). The affected epitopes were different in both studies, suggesting that LRRK2 signaling may affect different phospho‐sites within the same target proteins. Moreover, the overlapping DPPs related to LRRK2 mutation in G2019S carriers in our study and to LRRK2 enzymatic inhibition in healthy controls can hold interest as candidate pharmacodynamic biomarkers for LRRK2 inhibition in trials.

Despite some exciting observations on differences in signatures in LRRK2‐manifesting and nonmanifesting carriers, our study has limitations. We assessed posttranscriptional protein modification by phosphorylation mainly at Ser and Thr residues and Tyr residues, but not at noncanonical residues.^69^ The relationship between phosphorylated residues at the human phospho‐proteome is Ser/Thr/Tyr of 1800:200:1,^70^ but the number of the known canonical phosphorylated residues are estimated at 10^5^ and their phosphorylation status is rapidly dynamic.^71^ Second, we focused on the most abundant soluble fraction of the proteome, but screening hydrophobic transmembrane proteins encoded in the genome (25%)^72^ is technically challenging and requires alternative isolation methods. Third, in line with G2019S neutrophils,^22^ in G2019S PBMCs we did not detect differential T73 RAB10 phosphorylation. Fourth, as indicated for our isobaric label–based MS platform, we used group pooled samples, and accordingly, conclusions drawn from this study are to be interpreted only at the group level. Further establishment of clinical correlates of DPPs at the individual level requires larger studies addressing samples individually^50^ and using alternative platforms, such as label‐free data‐independent‐acquisition (DIA)/ sequential window acquisition of all theoretical mass spectra (SWATH‐MS).^73^

In summary, we performed an unbiased phospho‐proteomic screening in PBMCs from a large G2019S LRRK2 cohort. We uncovered a differential phospho‐signature associated with the G2019S mutation, which changes between asymptomatic and symptomatic G2019S carriers. Compliant with disease status, asymptomatic G2019S carriers were more similar to healthy controls than patients with G2019S L2PD. By identifying phospho‐protein candidates related to LRRK2 G2019S, our study contributes to understanding the biological pathways of mutant LRRK2 and helps decipher the dynamic molecular changes occurring before and after phenoconversion. We also generated DEP and DPP datasets from G2019S carriers, which we provide publicly to other researchers for data mining and phospho‐signature validation. If validated at the individual level in future studies, our findings may hold implications for disease prediction, patient enrichment for clinical trials, and early PD detection in PD at‐risk G2019S carriers. Further MS‐based phospho‐proteomic studies in larger G2019S populations are warranted.

## Financial Disclosures

E.T. received honoraria for consultancy from TEVA, Bial, Prevail, Boehringer Ingelheim, Roche, and BIOGEN.

M.F. was funded by the María de Maeztu program (grant MDM‐2017‐0729 to the Parkinson's Disease and Movement Disorders group of the Institut de Neurociènices [Universitat de Barcelona]).

R.F.‐S. was supported by a JIN grant of the MINECO and the AEI (AEI/FEDER/UE) (grant SAF2015‐73508‐JIN) and a Miguel Servet grant from the Instituto de Salud Carlos III (grant CP19/00048).

R.F.‐S., M.E., C.M., and E.S. were supported by The Michael J. Fox Foundation for Parkinson's Research (MJFF) (grant MJFF‐000858).

## Author Contributions

Conception and design of the study: Conception (C.M., M.E., R.F.‐S.); Organization (C.M., M.E., R.F.‐S.); Execution (E.S., J.F.‐I., M.S., M.F.); Patient recruitment (A.G., C.S., D.O., E.T., M.‐J.M.).

Acquisition and analysis of data: Design (R.F.‐S., M.E., C.M.); Execution (E.S., J.F.‐I.); Interpretation (A.G., E.S., J.F.‐I., E.T., M.‐J.M., S.P., C.M., M.E., R.F.‐S.); Review and Critique (all authors).

Drafting of the manuscript or figures: Writing of the first draft (R.F.‐S.); Draft editing (A.G., E.S., J.F.‐I., M.‐J.M., S.P., C.M., M.E., R.F.‐S.); Review and Critique (all authors).

## Supporting information


**Appendix S1.** Supporting Information

**FIGURE S1.** Volcano plot representations showing top DEPs and DPPs differences in comparisons involving G2019S L2PD and G2019S L2NMC, and controls based on fold‐change and *P*‐value criteria.
(**A**) Differentially expressed proteins (DEPs).
(**B**) Differentially phosphorylated proteins (DPPs).
Volcano plots were performed using default settings using VolcaNoSer software. Protein up‐regulation or hyper‐phosphorylation are depicted in red, and down‐regulation or hypo‐phosphorylation in blue. Annotated dots show the top‐10 candidates above thresholds (dashed line) and the largest (Manhattan) distance from the origin.
VolcaNoSer software: https://huygens.science.uva.nl/VolcaNoseR/.

**FIGURE S2.** Comparison of DEPs and DPPs among iPD, G2019S L2PD, and G2019S L2NMC compared to healthy controls as evaluated by Student *t* test (*P* < 0.05).
(**A**) Differentially expressed proteins (DEPs).
(**B**) Differentially phosphorylated proteins (DPPs).
In each comparison, the number and the lists of common or specific DEPs / DPPs are depicted in Venn diagrams and adjacent tables.

**FIGURE S3.** Biological enrichment analysis of DEPs and DPPs in G2019S L2PD, G2019S L2NMC, and controls detected by ANOVA multi‐group analysis.
(**A**) interlocked DEPs and DPPs.
(**B**) DEPs and DPPs per separate.
Statistically significant Gene Ontology (GO) terms were adjusted by multiple testing adjustment based MS of P‐values (adj. *P* < 0.05, dashed line) as analyzed using Metascape (Reactome database). This analysis showed similar pathway deregulation using interlocked DEPs and DPPs or DEPs and DPPs per separate in G2019S carriers.

**FIGURE S4.** Ingenuity functional network analysis of interlocked DEPs and DPPs from pairwise comparison showing distinctive networks shared by G2019S L2PD, G2019S L2NMC, and iPD.
(**A**) AKT‐centred network in G2019S L2PD, G2019S L2NMC, and iPD.
(**B**) NFKB‐centred network in G2019S L2PD, G2019S L2NMC, and iPD.

Table S1

Table S2

Table S3

Table S4

Table S5

Table S6

Table S7

Table S8

Table S9

Table S10

## Data Availability

MS data and search results files were deposited in the ProteomeXchange Consortium via the JPOST partner repository (https://repository.jpostdb.org)^74^ with the identifier PXD028221 for ProteomeXchange and JPST001313 for jPOST. For reviewers, please visit: https://repository.jpostdb.org/preview/181779812761373e0fc6d85 Access key: 9345
