# Supplementary material for: Differential Phospho‐Signatures in Blood Cells Identify LRRK2 G2019S Carriers in Parkinson's Disease
Source: Mov Disord. 2022 Jan 20;37(5):1004–15. doi: 10.1002/mds.28927 (PMC9306798; doi:10.1002/mds.28927)
Supplement: Supplementary file 1 — Appendix S1. Supporting Information [file MDS-37-1004-s007.docx]

**SUPPLEMENTARY METHODS**

**Reduction, alkylation, digestion, and tandem-mass-tag (TMT) labeling**

For proteomic analyses, we used 600 µg of protein from each pooled sample. Proteins were first reduced with 10mM TCEP for 1h at 30°C. Then, alkylation was performed 30 min at room temperature using 40mM IAA. Samples were diluted 5 times to reduce the urea concentration <1M, and protein enzymatic cleavage was carried out overnight with trypsin (Promega; 1:50, w/w) at 37°C. Subsequently, each pooled sample was labeled with one of the TMT reagents according to the manufacturer's instructions (Thermo Scientific). We performed a TMT experiment for the core analysis to monitor the experimental groups. Thus, control pools were labeled with 126 and 127 respectively (n=15/each), G2019S L2PD with 128 and 129 (n=10/each), and G2019S L2NMC with 130 and 131 (n=10/each). In the second TMT experiment involving exploratory analyses, control pools were labelled with 126 (n=15), iPD with 127 (n=15), G2019S L2PD with 128 (n=10), G2019S L2NMC with 130 (n=10), R1441G L2PD with 129 (n=5), and R1441G L2NMC with 131 (n=3). After incubating the labeling reaction for 60 min, all samples included in each TMT assay were pooled and dried in a speed vacuum.

**Chromatographic and MS parameters**

Unbound peptide pools (non-modified peptides) were dried in a vacuum centrifuge and reconstituted with 40 µl of 5 mM ammonium bicarbonate (ABC) pH 9.8 and injected into an ÄKTA pure 25 System (GE Healthcare Life Sciences) with a high pH stable X-Terra RP18 column (C18; 2.1 mm × 150 mm; 3.5 μm) (Waters). Mobile phases were 5mM ammonium formate in 90% ACN at pH 9.8 (buffer B) and 5mM ammonium formate in water at pH 9.8 (buffer A). Column gradients were developed in an 80 min three-step gradient (from 5% B to 30% B in 5 min, 30% B to 60% B in 40 min, 15 min in 60% B, and 60% B to 90% B in 20 min). The column was equilibrated in 95% B for 30 min and 2% B for 10 min. 8 fractions were collected and evaporated under a vacuum. Peptide fractions were reconstituted into a final concentration of 0.5µg/µL of 2% ACN, 0.5% FA, 97.5% MilliQ-water before mass spectrometric analysis. Then, peptides mixtures were separated by reverse phase chromatography using an Eksigent nanoLC ultra 2D pump fitted with a 75 μm ID column (Eksigent 0.075 x 250). Samples were first loaded for desalting and concentration into a 2 cm length 100 μm ID precolumn packed with the same chemistry as the separating column. Mobile phases were 100% water 0.1% formic acid (FA) (buffer A) and 100% Acetonitrile 0.1% FA (buffer B). Non-modified peptide fractions were analyzed following the next conditions. Column gradient was developed in a 135 min three-step gradient (from 2% B to 30% B in 90 min, from 30% B to 40% B in 10 min, and from 40% to 80% in 10 min). The column was equilibrated in 97% B for 3 min and 2% B for 23 min. During all processes, precolumn was in line with the column, and flow was maintained during gradient at 300 nl/min. Eluting peptides from the column were analyzed using a 5600 Triple-TOF system (Sciex). Information data was acquired upon a survey scan performed in a mass range from 350 m/z up to 1250 m/z in a scan time of 250 ms. Top-35 peaks were selected for fragmentation. The minimum accumulation time for MS/MS was set to 11 ms giving a total cycle time of 3.8 s. Productions were scanned in a mass range from 100 m/z to 1500 m/z and excluded for further fragmentation during 15 s. In the case of fractions containing the phosphorylated peptides, column gradient was developed in a 140 min two-step gradient from 2% to 35% B in 100 min and from 35% to 70% in 20 min. The column was equilibrated in 95% B for 5 min and 2% B for 15 min. Precolumn was in line with the column, and flow was maintained along the gradient at 300 nl/min. Again, eluting peptides from the column were analyzed using a 5600 Triple-TOF system (Sciex) following the same conditions as the non-modified peptides.
