# Supplementary figures and images for: Differential Phospho‐Signatures in Blood Cells Identify LRRK2 G2019S Carriers in Parkinson's Disease
Source: Mov Disord. 2022 Jan 20;37(5):1004–15. doi: 10.1002/mds.28927 (PMC9306798; doi:10.1002/mds.28927)

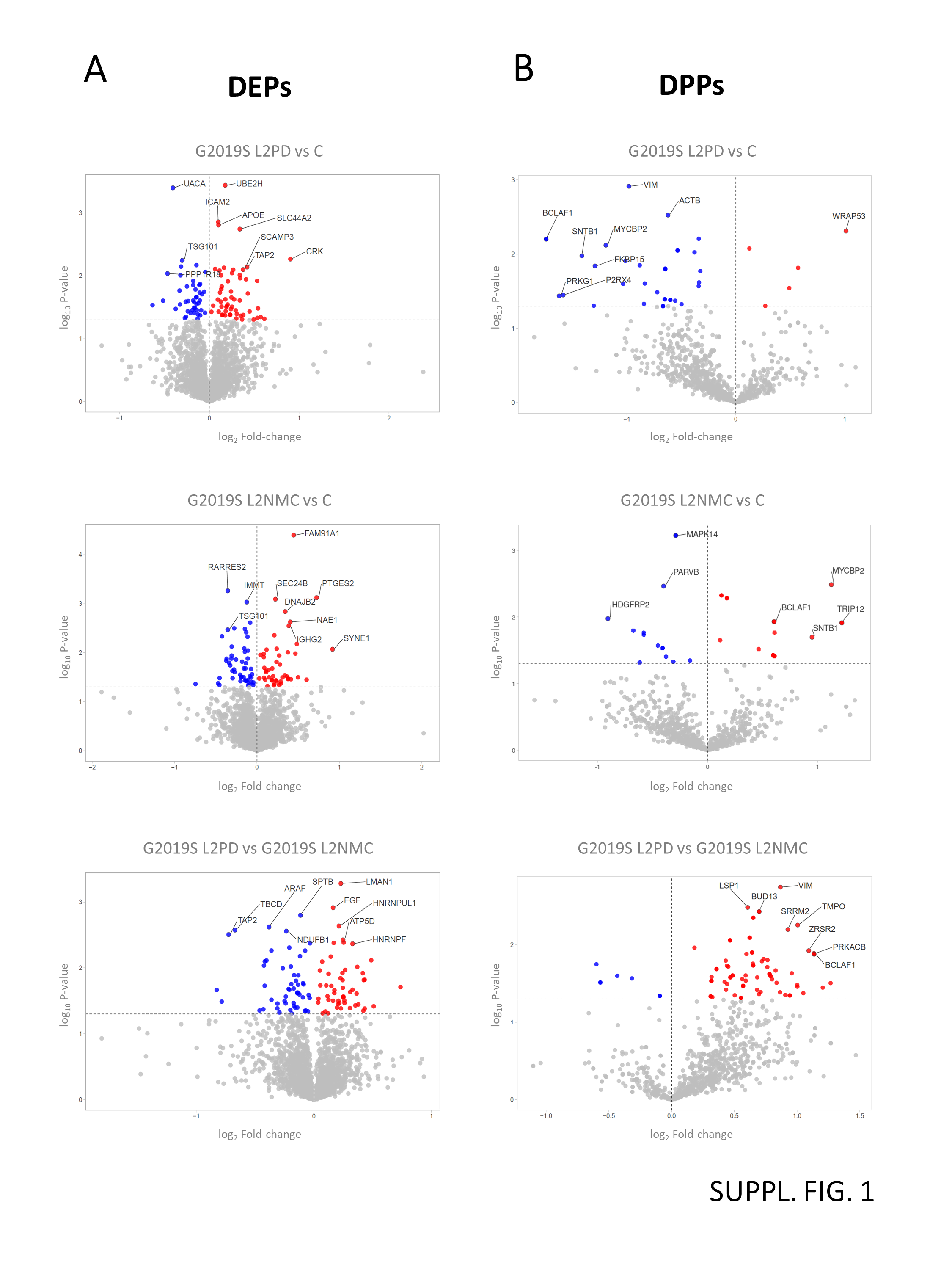

Supplement: Supplementary file 2 — FIGURE S1. Volcano plot representations showing top DEPs and DPPs differences in comparisons involving G2019S L2PD and G2019S L2NMC, and controls based on fold‐change and P‐value criteria. (A) Differentially expressed proteins (DEPs). (B) Differentially phosphorylated proteins (DPPs). Volcano plots were performed using default settings using VolcaNoSer software. Protein up‐regulation or hyper‐phosphorylation are depicted in red, and down‐regulation or hypo‐phosphorylation in blue. Annotated dots show the top‐10 candidates above thresholds (dashed line) and the largest (Manhattan) distance from the origin. VolcaNoSer software: https://huygens.science.uva.nl/VolcaNoseR/. [file MDS-37-1004-s003.tif]

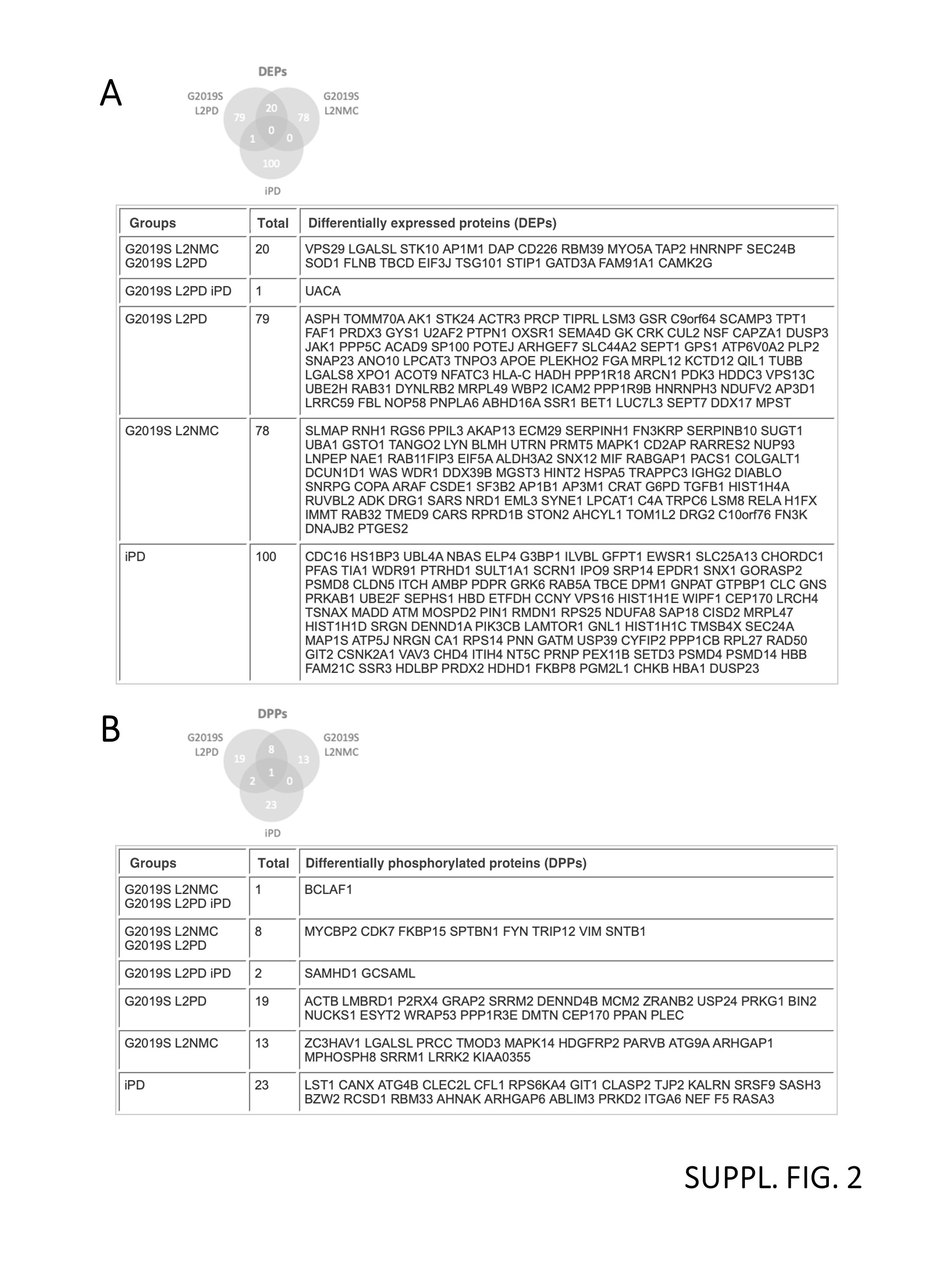

Supplement: Supplementary file 3 — FIGURE S2. Comparison of DEPs and DPPs among iPD, G2019S L2PD, and G2019S L2NMC compared to healthy controls as evaluated by Student t test (P < 0.05). (A) Differentially expressed proteins (DEPs). (B) Differentially phosphorylated proteins (DPPs). In each comparison, the number and the lists of common or specific DEPs / DPPs are depicted in Venn diagrams and adjacent tables. [file MDS-37-1004-s012.tif]

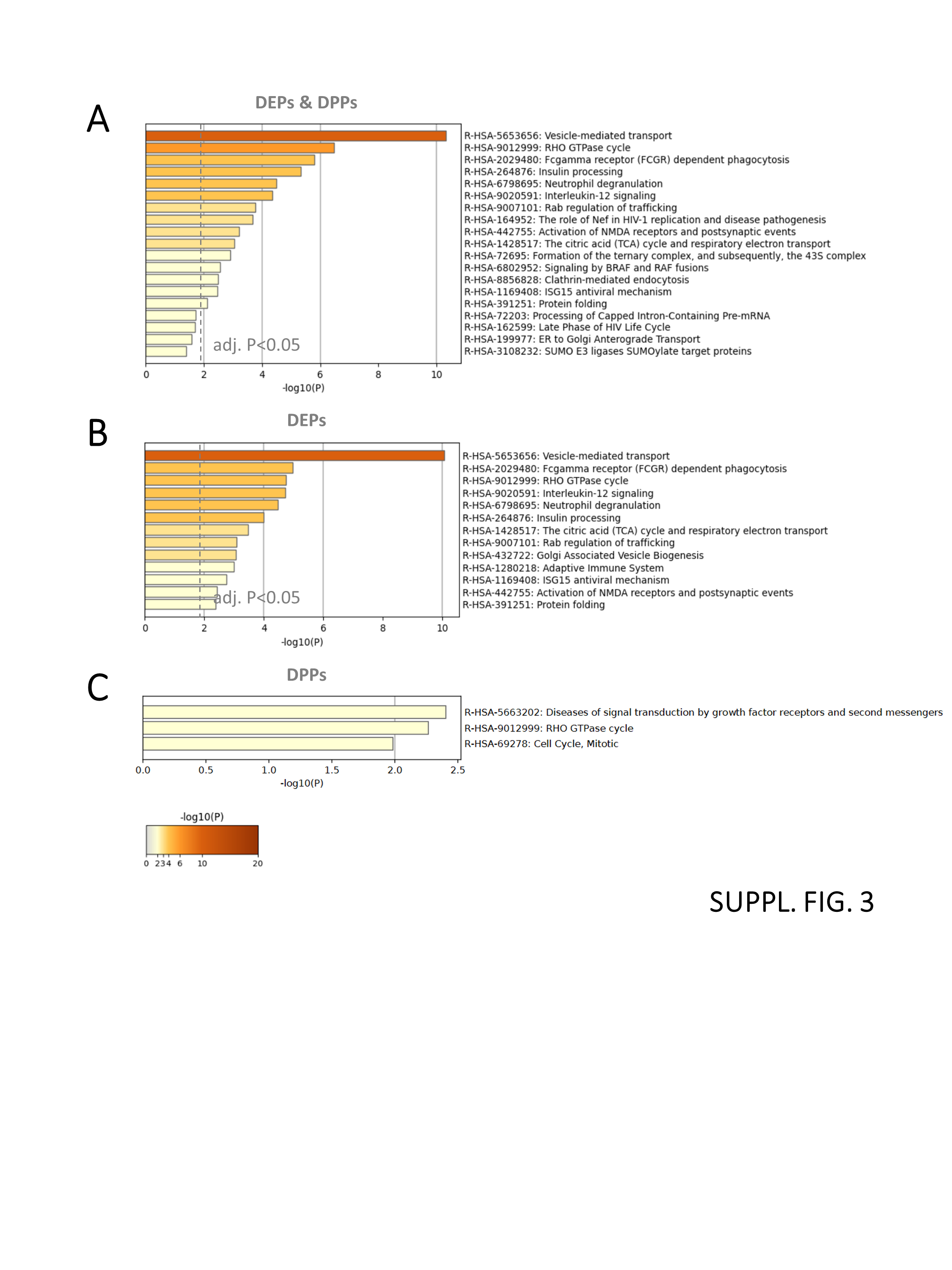

Supplement: Supplementary file 4 — FIGURE S3. Biological enrichment analysis of DEPs and DPPs in G2019S L2PD, G2019S L2NMC, and controls detected by ANOVA multi‐group analysis. (A) interlocked DEPs and DPPs. (B) DEPs and DPPs per separate. Statistically significant Gene Ontology (GO) terms were adjusted by multiple testing adjustment based MS of P‐values (adj. P < 0.05, dashed line) as analyzed using Metascape (Reactome database). This analysis showed similar pathway deregulation using interlocked DEPs and DPPs or DEPs and DPPs per separate in G2019S carriers. [file MDS-37-1004-s010.tif]

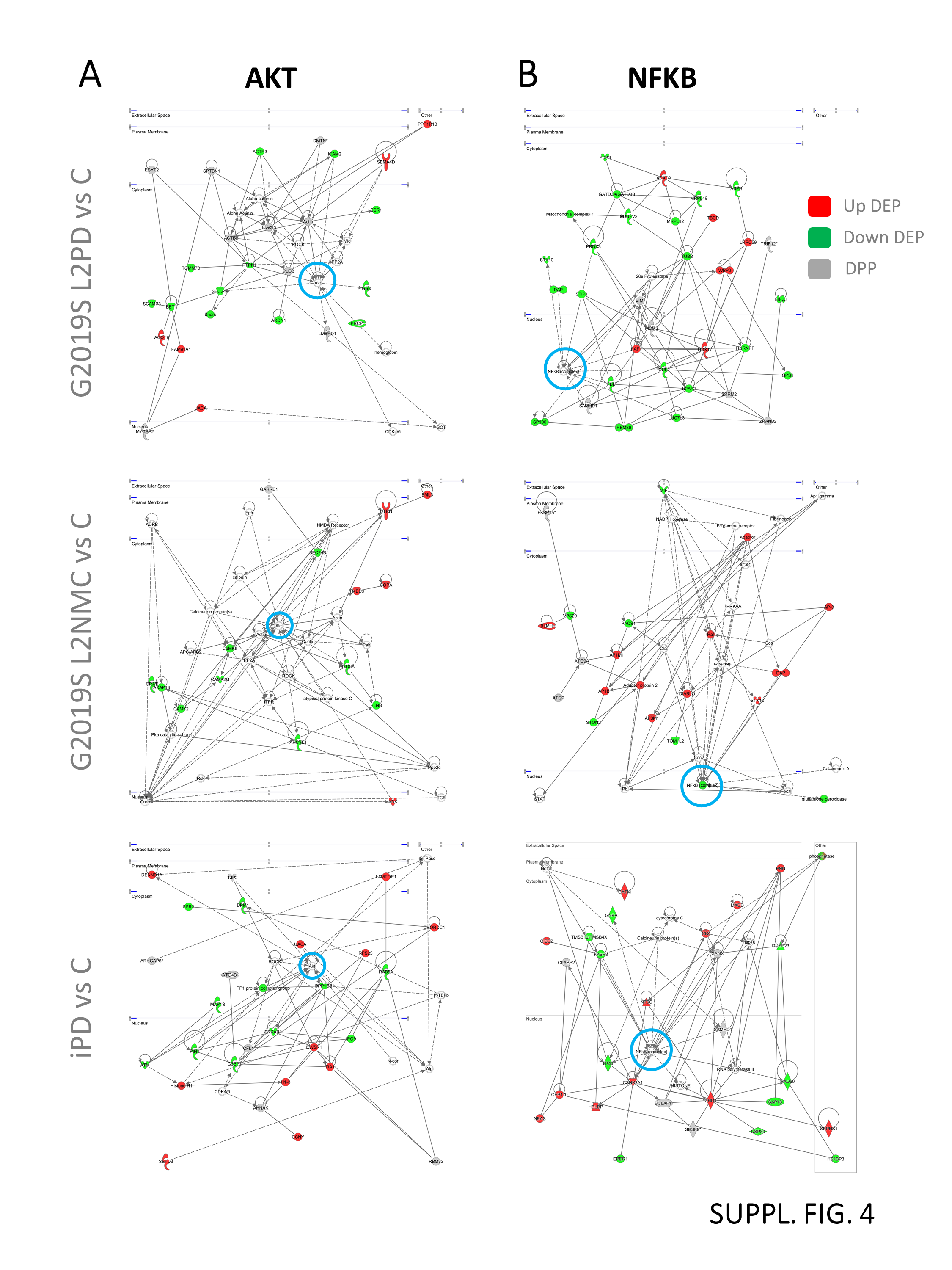

Supplement: Supplementary file 5 — FIGURE S4. Ingenuity functional network analysis of interlocked DEPs and DPPs from pairwise comparison showing distinctive networks shared by G2019S L2PD, G2019S L2NMC, and iPD. (A) AKT‐centred network in G2019S L2PD, G2019S L2NMC, and iPD. (B) NFKB‐centred network in G2019S L2PD, G2019S L2NMC, and iPD. [file MDS-37-1004-s006.tif]
